# The association between genetic variants in *hMLH1 *and *hMSH2 *and the development of sporadic colorectal cancer in the Danish population

**DOI:** 10.1186/1471-2350-9-52

**Published:** 2008-06-11

**Authors:** Lise Lotte Christensen, Bo E Madsen, Friedrik P Wikman, Carsten Wiuf, Karen Koed, Anne Tjønneland, Anja Olsen, Ann-Christine Syvänen, Claus L Andersen, Torben F Ørntoft

**Affiliations:** 1 Molecular Diagnostic Laboratory, Department of Clinical Biochemistry, Aarhus University Hospital, Skejby, Denmark; 2 Bioinformatics Research Center (BiRC), University of Aarhus, Denmark; 3 Faculty of Medical Laboratory Technology, University College Jutland, Aarhus, Denmark; 4 Danish Cancer Society, Institute of Cancer Epidemiology, Copenhagen, Denmark; 5 Department of Medical Sciences, Uppsala University Hospital, Sweden

## Abstract

**Background:**

Mutations in the mismatch repair genes *hMLH1 *and *hMSH2 *predispose to hereditary non-polyposis colorectal cancer (HNPCC). Genetic screening of more than 350 Danish patients with colorectal cancer (CRC) has led to the identification of several new genetic variants (e.g. missense, silent and non-coding) in *hMLH1 *and *hMSH2*. The aim of the present study was to investigate the frequency of these variants in *hMLH1 *and *hMSH2 *in Danish patients with sporadic colorectal cancer and in the healthy background population. The purpose was to reveal if any of the common variants lead to increased susceptibility to colorectal cancer.

**Methods:**

Associations between genetic variants in *hMLH1 *and *hMSH2 *and sporadic colorectal cancer were evaluated using a case-cohort design. The genotyping was performed on DNA isolated from blood from the 380 cases with sporadic colorectal cancer and a sub-cohort of 770 individuals. The DNA samples were analyzed using Single Base Extension (SBE) Tag-arrays. A Bonferroni corrected Fisher exact test was used to test for association between the genotypes of each variant and colorectal cancer. Linkage disequilibrium (LD) was investigated using HaploView (v3.31).

**Results:**

Heterozygous and homozygous changes were detected in 13 of 35 analyzed variants. Two variants showed a borderline association with colorectal cancer, whereas the remaining variants demonstrated no association. Furthermore, the genomic regions covering *hMLH1 *and *hMSH2 *displayed high linkage disequilibrium in the Danish population. Twenty-two variants were neither detected in the cases with sporadic colorectal cancer nor in the sub-cohort. Some of these rare variants have been classified either as pathogenic mutations or as neutral variants in other populations and some are unclassified Danish variants.

**Conclusion:**

None of the variants in *hMLH1 *and *hMSH2 *analyzed in the present study were highly associated with colorectal cancer in the Danish population. High linkage disequilibrium in the genomic regions covering *hMLH1 *and *hMSH2*, indicate that common genetic variants in the two genes in general are not involved in the development of sporadic colorectal cancer. Nevertheless, some of the rare unclassified variants in *hMLH1 *and *hMSH2 *might be involved in the development of colorectal cancer in the families where they were originally identified.

## Background

Colorectal cancer (CRC) is a common malignant disease in the western world. The lifetime risk is about 5% and rising [1]. In 2003, approximately 3,600 new cases where registered by the Danish Cancer Registry equivalent to 10% of the total number of malignant cancer cases in Denmark, making CRC the third most common cancer in the country [2].

A twin study has demonstrated that up to 35% of the CRCs can be explained by inherited susceptibility [3]. However, only approximately 5% of the CRCs are explained by well defined hereditary syndromes displaying a Mendelian inheritance pattern. The most common form of hereditary CRC is hereditary non-polyposis colorectal cancer (HNPCC) [4,5]. Diagnosis of HNPCC is based on kindred analysis using the Amsterdam II criteria [6]. In the major part of the HNPCC families the disease is caused by loss of function pathogenic mutations such as nonsense, frameshift and non-coding mutations affecting splice sites identified mainly in three genes i.e. *hMLH1*, *hMSH2 *and *hMSH6 *[7].

A part from the clearly pathogenic mutations genetic screening has also revealed numerous missense, silent and non-coding variants of unknown significance in *hMLH1*, *hMSH2 *and *hMSH6*. Ideally, segregation studies should be conducted to reveal the pathogenicity of a given variant. However, such analyses are often not feasible due to limited family sizes and unavailability of clinical specimens. Consequently, different evaluation methods have been used, especially for the missense variants, to be able to distinguish neutral variants from disease-causing mutations. Functional analyses of individual missense variants have been carried out using different *in vitro *assays e.g. [8-13]. The functional studies have revealed both loss of function mutations that are most likely pathogenic [8-13] and variants with reduced activity [8-11]. Apart from non-coding mutations affecting the classical splice sites at intron/exon junctions, missense changes as well as silent changes in *hMLH1 *and *hMSH2 *have also been shown to alter pre-mRNA splicing and thereby causing exon skipping [14,15]. Functional analyses are laborious and therefore *in silico *methods available on the internet (e.g. SIFT, PolyPhen and PMUT) have be used to identify variants that should be selected for further functional analysis [16].

*In silico *predictions and *in vitro *functional analyses provide an idea of which variants are pathogenic. However, population studies are needed to reveal the *in vivo *pathogenicity of the individual variants. At present, very few population studies have dealt with the association of common variants in *hMLH1 *and *hMSH2 *with susceptibility to sporadic CRC [17-19].

The present study describes a population based analysis of the frequency of variants of unknown significance in *hMLH1 *and *hMSH2 *in Danish patients with sporadic CRC and in a sub-cohort of controls. Some of the variants have been described in other studies, and some are, to our knowledge, new variants unique to the Danish population. We have analyzed the allele frequencies of 29 variants in *hMLH1 *and *hMSH2 *to reveal whether they cause increased susceptibility to sporadic CRC in the Danish population or whether they are private variants/mutations found only in the families where they were originally identified. In addition to the 29 variants identified in Danish individuals, six variants identified in other Caucasian populations were also investigated.

## Methods

### Subjects/cohort

The subjects were selected from the Danish Diet, Cancer and Health (DCH) study, which is an ongoing prospective follow-up study [20]. In all 57,053 men and women born in Denmark, living in the greater Copenhagen or Aarhus areas, aged 50–64, and with no previous cancer diagnosis at the time of enrolment, were included in the study. The participants were recruited during the years 1993–1997. At the time of enrolment, biological material from blood, urine, nails and fat tissue was sampled and stored in liquid nitrogen at -150°C. Among the cohort members 380 cases with CRC diagnosed between 1994 and 2004 were identified in the files of the Danish Cancer Registry. A sub-cohort of 770 controls (including 10 cases) was selected randomly from the cohort. The characteristics of the cohort are shown in Table 1. General protocols concerning the Diet, Cancer and Health study have been evaluated and approved by the Regional Scientific Committees on Human Studies in Copenhagen and Aarhus (journal number: H-KF-01-345/93) and the Danish Data Protection Agency. A protocol regarding the present study has been submitted and approved by the mentioned committees as a supplement to the initial protocols.

**Table 1:** Characteristics of the cohort

	Cases (n = 380)	Sub-cohort (n = 770)
Sex (Number (%))		
Men	213 (56%)	427 (55%)
Women	167 (44%)	343 (45%)
Age* (Years (sd))		
Men	58.4 (4.1)	56.9 (4.5)
Women	58.5 (4.5)	56.4 (4.4)
Time observed (Years (sd))		
Men	-	6.6 (1.2)
Women	-	6.6 (1.0)

*Age at inclusion into the cohort given as mean (sd) in years

Another cohort named familiar CRC consisting of 285 CRC cases was also included in the study. The majority of the variants were initially identified in this cohort. Some variants were found only in one family whereas others were identified in several families in the cohort. The cohort consists of individuals from HNPCC families (based on the Amsterdam II criteria) or individuals from families not fulfilling the Amsterdam II criteria, but with a clear familial accumulation of CRC. The presence of other clearly pathogenic mutations in the *hMLH1*, *hMSH2 *and *hMSH6 *has been excluded by sequencing. All individuals in the cohort of familiar CRC have been consulted by a clinical geneticist and informed consent was obtained from all individuals in the cohort. Detailed information on each individual family in this cohort is not available.

### DNA samples

DNA was extracted from frozen leukocytes as described previously [21].

### Variants in *hMLH1 *and *hMSH2*

Initially, 47 variants in *hMLH1 *and *hMSH2 *were included in the study. Forty-one of the variants have previously been identified either in Danish HNPCC patients (i.e. fulfilling the Amsterdam II criteria) or in patients not fulfilling the Amsterdam II criteria but with a familial accumulation of CRC. Six variants have been identified in other Caucasian populations. Of the initial 47 selected variants, 12 variants were excluded either due to troubles regarding the primer design or lack of amplification product in the multiplex PCR. The remaining 35 analyzed variants are listed in Table 2.

**Table 2:** *MLH1 *and *MSH2 *variants

Id	Variant	Amino acid change	Identified in the present study	Phylogeny	Pathogenic status	References
	*MLH1*					
23	c.307-29 C>A	Intronic	+	-	-	-
24	c.350 C>T	p.Thr117Met	-	Conserved	Pathogen	[11,50]
25	c.453+79 A>G	Intronic	+	-	-	-
26	c.545+43 C>G	Intronic	+	-	-	-
27	c.655 A>G	p.Val219Ile	+	Ile other species	Neutral	[11,40,51]
28	c.790+10 A>G	Intronic	+	-	-	-
29	c.884+39 G>A	Intronic	-	-	-	-
30	c.884+83_84 ins T	Intronic	-	-	-	-
85	c.1217 G>A	p.Ser406Asn	+	Non-conserved	Neutral	[11,41,52]
31	c.1379 A>C	p.Glu460Ala	-	Non-conserved	-	-
86*	c.1558+11 G>A	Intronic	-	-	Neutral	[53]
32	c.1558+14 G>A	Intronic	+	-	Neutral	[40]
37	c.1668-19 A>G	Intronic	+	-	Neutral	[40]
101	c.1689 A>G	p.Ile563Met	-	Non-conserved	-	-
33	c.1732-2 A>T	Intronic	-	-	Pathogen	[54]
34	c.1852_1853 AA>GC	p.Lys618Ala	+	Non-conserved	Neutral/Pathogen	[9,11,45,46,48]
100	c.1942 C>T	p.Pro648Ser	-	Conserved	Pathogen	[9,45,55]
35	c.1959 G>T	p.Leu653Leu	+	-	Neutral	[42]
36	c.2152 C>T	p.His718Tyr	-	Conserved	Neutral	[11,56]
						
	*MSH2*					
89	c.-118 T>C	promoter	+	-	-	[32,33]
87	c.131 C>T	p.Thr44Met	-	Conserved	Pathogen	[55]
88	c.134 C>T	p.Ala45Val	-	Val other species	Neutral	[55]
39	c.212-23 A>C	Intronic	-	-	-	-
90*	c.287 G>A	p.Arg96His	-	Non-conserved	Neutral	[57,58]
91*	c.329 A>G	p.Lys110Arg	-	Non-conserved	Neutral	[57]
92*	c.380 A>G	p.Asn127Ser	-	Conserved	Neutral/Pathogen	[48,59]
93	c.560 T>G	p.Leu187Arg	-	Conserved	Neutral/Pathogen	[60]
41	c.965 G>A	p.Gly322Asp	+	Conserved	Neutral	[51,61]
43	c.1511-9 A>T	Intronic	+	-	-	-
48	c.1786_1788 del AAT	p.Asn596del	-	Non-conserved	Pathogen	[62,63]
95*	c.2006-6 T>C/G	Intronic	-	-	Neutral	[64]
96	c.2062 A>G	p.Met688Val	-	Conserved	-	-
97*	c.2139 G>C	p.Gly713Gly	-	-	Neutral	[57,65]
50	c.2500 G>A	p.Ala834Thr	-	Non-conserved	Neutral	[10,66]
51	c.2542 G>T	p.Ala848Ser	-	Conserved	-	-

* identified in CRC families from other Caucasian populations than the Danish

### Genotyping using Single Base Extension (SBE)-tag arrays

#### Primer design

The multiplex PCR primers were designed using Oligo 6 [22]. The PCR products were between 100 and 450 bp. The optimal annealing temperature (Ta) was set to ~50°C, the GC content to ~40% and the primer Tm difference to ≤1. The single base extension (SBE) primers contained 5' 20-bp 'tag' sequences that were from the Affymetrix GeneChip^® ^Tag Collection. The gene specific part of the SBE primers had a Tm of ~50–60°C (calculated using PrimerExpress 2.0)(Applied Biosystems, Foster City, CA). The tagged SBE primers were tested for major hairpin loop formation using Oligo6. All variants were typed using SBE-primers for both DNA strands. The primers were synthesized by DNA Technology (Aarhus, Denmark). The multiplex PCR primer and the SBE primer sequences are available on request.

#### Multiplex PCR

The multiplex PCR amplification was performed with 6–9 primer pairs per reaction. Twenty ng of genomic DNA with 0.5 μl Accuprime™ DNA polymerase (Invitrogen, Carlsbad, CA), 1× Accuprime™ Buffer I, and 0.08–1 μM primers was amplified in 25 μl volumes using the following PCR conditions: 1 cycle of 95°C for 10 min; 13 cycles of 95°C for 30 sec., 67°C (-1°C/cycle) for 45 sec. and 72°C for 45 sec.; 20 cycles of 95°C for 30 sec., 55°C for 45 sec. and 72°C for 45 sec.; 1 cycle of 72°C for 10 min. A subset of the multiplex PCR products were analysed on a 2100 Bioanalyzer (Agilent Technologies, Santa Clara, CA) to test the performance of the multiplex PCR. The multiplex PCR products from each sample were pooled for further analysis.

#### Single Base Extension (SBE) reaction

The pooled PCR products from each sample were treated with Exonuclease I and shrimp alkaline phosphatase and used for template in the SBE reaction (mini-sequencing) as described by Lindross et al. [23]. The fluorescent labelled ddNTP were Cyanine 5 (Cy5)-ddCTP, TAMRA-ddUTP, Texas Red^®^-5-ddATP and R110-ddGTP (PerkinElmer Life and Analytical Sciences, Inc, Wellesley, Ma).

#### Preparation of microarrays

'Anti-tag' oligonucleotides complimentary to the 'tag' sequences of the SBE primers modified with NH_2 _groups in their 3' ends, and containing a 3'-spacer of 15 T residues were coupled covalently to CodeLink Activated Slides (Amersham Biosciences, Uppsala, Sweden) according to manufacturer instructions. The only exception was that the 'anti-tag' oligonucleotides were dissolved in 150 mM sodium carbonate buffer, pH 8.5 with 1 mM betaine at a concentration of 20 μM. The oligonucleotides were printed onto the slides using a VersArray ChipWriter (BioRad, Hercules, CA) with 3 Stealth Micro Spotting Pins (TeleChem International Inc., Sunnyvale, CA). Each slide consists of 75 sub-arrays with 13 × 12 spots in each subarray. The spots were 130–150 μM in diameter and the centre-to-centre distance between two spots was 200 μM. The 'anti-tag' oligonucleotides were printed in duplicates in each sub-array. The spot quality was tested after each series of microarray preparation using a Cy3 labelled random oligonucleotide hybridizing to all spots on the slide independent on the 'anti-tag' sequence. The printed slides were stored at room temperature until use.

#### Hybridization of the SBE reaction products

The slides printed with 'anti-tags' were pre-heated to 42°C in a custom-made aluminium reaction rack with a re-usable silicon rubber grid placed on the slides to form 75 separate reaction chambers on each slide [24]. The hybridization mixtures, containing the SBE reaction products in 6.5 × SSC were added to each reaction chamber on the pre-heated slide. The hybridization time was 2.5 hours at 42°C followed by a brief rinse with 4 × SSC at room temperature. Subsequently, the slides were washed twice for 5 min. in 2 × SSC, 0.1% SDS (42°C) and twice for 1 min. in 0.2 × SSC (room temperature). Finally, the slides were spin dried for 3 min. at 800 rpm.

#### Signal detection

The signal detection was performed mainly as described by Lindroos et al. [23]. The fluorescence signals were detected with a ScanArray 4000XL instrument and the ScanArray Express software (PerkinElmer Life and Analytical Sciences, Inc, Wellesley, Ma). The four excitation lasers were: blue Argon, 488 nm; Green HeNe, 543.8 nm; Yellow HeNe, 594 nm and Red HeNe 632.8 nm. The laser power was kept constant at 90%, whereas the photo-multiplier tube (PMT) varied between fluorophores. A typical setting for the PMT gain was 65, 75, 65 and 70% for the fluorophores Cy5-ddCTP, TAMRA-ddUTP, Texas Red^®^-5-ddATP and R110-ddGTP, respectively.

### Statistics

#### Association tests

A Bonferroni corrected Fisher exact test was used to test for association between the genotypes of each SNP and the familiar CRC cohort and the cohort of sporadic CRC, respectively. Fifteen tests were made for single marker associations, in the cohort of familiar CRC, yielding a Bonferroni corrected significance level of 0.05/15 = 0.0033. Likewise the Bonferroni corrected *p*-value of the cohort of sporadic cases was 0.05/13 = 0.0038. A Bonferroni corrected Monte Carlo (MC) Fisher exact test was used to test for association between each pair of SNPs, and sporadic or familiar CRC. One hundred thousand permutations were used for each test. Missing data were handled by omitting persons with missing data in the genotype counts; e.g. all individuals with missing data in either variant id 26 or variant id 27 were omitted when calculating the genotype frequencies of that specific SNP-pair.

#### Power calculations

The power was calculated by applying the Fisher exact test to 10000 independent simulated cases with the given odds ratio and frequency of the disease causing genotype. In that case the power is the proportion of the simulations that reach a *p*-value lower than 0.05/15 = 0.0033.

#### Test for Hardy-Weinberg proportions

Each variant was tested for deviation from Hardy-Weinberg proportions in the sub-cohort, using the exact test described by Wigginton et al. [25]. The used significance level was 0.01.

### Linkage disequilibrium

Linkage disequilibrium (LD) was investigated using HaploView (v. 3.31), [26] and genome build NCBI35 for information track.

### In *silico *analysis

In *silico *prediction of the functional consequence of the missense variants was performed using SIFT (Sorting Intolerant From Tolerant) [27], Polyphen [28] and PMUT [29]. Mis-splicing was analysed using SNAP (SNP Annotation Platform) [30]. The alignments of MSH2 or MLH1 polypeptides used in the phylogenetic analysis of the missense variants was performed using ClustalW [31].

## Results

### Performance of the Single Base Extension-tag arrays

Single base extension (SBE)-tag arrays is a well described method for analysing single nucleotide polymorphisms [23]. Initially, 47 variants were included in the study. However, 12 variants either failed the initial primer design or they did not perform well in the multiplex PCR reaction. The accuracy of the SBE-tag array in the genotyping of the remaining 35 variants was tested using samples with known genotypes. All tested samples were correctly genotyped (data not shown). A total of 31 of 35 variants performed very well in the assay, i.e. more than 70% of the samples had positive call (data not shown).

### Frequency of variants in the three analysed cohorts

We have identified the frequency of 35 variants in *hMLH1 *and *hMSH2 *in a well defined cohort of 770 individuals representative of the Danish population. In addition, the frequency of the variants was also identified in 380 cases with sporadic CRC and in 285 individuals with familiar CRC. The analyzed variants were initially identified in the familiar CRC cohort and the cohort was included in the study to elucidate whether a common variant could explain the elevated cancer susceptibility in these families.

The characteristics of the cohort of sporadic CRC cases and the sub-cohort are shown in Table 1. Thirteen out of 35 variants were polymorphic in the cohort of sporadic CRC cases and/or in the sub-cohorts (Tables 2 and 3). Ten of these variants were detected with a frequency ≥1% in the sub-cohort and are therefore relatively common in the Danish population. The remaining three variants were present with a frequency <1% (Table 3). None of the analysed variants deviated significantly from the Hardy-Weinberg equilibrium. Out of the 13 identified variants 9 were non-coding or silent variants and 3 were non-conserved missense changes and one was a conserved missense change (Table 2). The frequency of 11/13 variants did not differ significantly between any of the three analyzed cohorts neither individually (Table 4) nor as pairs (data not shown). The frequency of one variant; *hMSH2 *c.-118 T>C differed significantly between the sporadic CRC cases and the sub-cohort with a borderline significant *p*-value of 0.0037 (significance level: 0.05/13 = 0.0038). One previous study has demonstrated a slight difference between the frequencies of this variant in HNPCC cases and controls (*p*-value of 0.034) [32], whereas another study detected no difference [33]. In addition, it has also been shown that the variant does not change the promoter activity of the *hMSH2 *promoter *in vitro *and it is therefore most likely not involved in increased susceptibility to CRC [32]. The frequency of the variant *hMLH1 *c.1668-19 A>G differed between the familiar CRC cases and the sub-cohort with a borderline significant *p*-value of 0.0044 (significance level: 0.05/15 = 0.0033). This variant is an intronic variant which has never been characterized neither *in vivo *nor *in vitro*. *In silico *analyses using SNAP (a SNP Annotations Platform) [30] demonstrated that the presence of this variant results in the elimination of an Exon Splicing Enhancer (ESE) (SRp40) and the introduction of a new ESE (SRp55) (data not shown). However, *in vitro *analysis must be performed to reveal if these changes result in an aberrant splice pattern.

**Table 3:** Genotype frequencies of the variants in the analyzed cohorts

Id	Variant	Sporadic cases			Familiar CRC cohort			Sub-cohort		
	*MLH1*	Ho_Wt_	He	Ho_Mut_	Ho_Wt_	He	Ho_Mut_	Ho_Wt_	He	Ho_Mut_
		
23	**c.307-29 C>A**	**0.994**	**0.006**	**0.000**	**0.990**	**0.010**	**0.000**	**0.990**	**0.010**	**0.000**
24	c. 350 C>T	1.000	0.000	0.000	0.997	0.003	0.000	1.000	0.000	0.000
25	**c.453+79 A>G**	**0.276**	**0.482**	**0.242**	**na**^§^	**na**	**na**	**0.297**	**0.465**	**0.238**
26	**c.545+43 C>G**	**0.997**	**0.003**	**0.000**	1.000	0.000	0.000	1.000	0.000	0.000
27	**c.655 A>G**	**0.451**	**0.448**	**0.101**	**0.492**	**0.427**	**0.081**	**0.472**	**0.425**	**0.102**
28	**c.790+10 A>G**	**0.994**	**0.006**	**0.000**	**na**	**na**	**na**	**0.991**	**0.009**	**0.000**
29	c.884+39 G>A	1.000	0.000	0.000	0.997	0.003	0.000	1.000	0.000	0.000
30	c.884+83_84 ins T	1.000	0.000	0.000	1.000	0.000	0.000	1.000	0.000	0.000
85	**c.1217 G>A**	1.000	0.000	0.000	**0.997**	**0.003**	**0.000**	**0.998**	**0.002**	**0.000**
31	c.1379 A>C	1.000	0.000	0.000	0.994	0.006	0.000	1.000	0.000	0.000
86*	c.1558+11 G>A	1.000	0.000	0.000	1.000	0.000	0.000	1.000	0.000	0.000
32	**c.1558+14 G>A**	**0.992**	**0.072**	**0.000**	**0.919**	**0.081**	**0.000**	**0.928**	**0.072**	**0.000**
37	**c.1668-19 A>G**	**0.292**	**0.504**	**0.204**	**0.372**	**0.485**	**0.142**	**0.305**	**0.469**	**0.226**
101	c.1689 A>G	1.000	0.000	0.000	1.000	0.000	0.000	1.000	0.000	0.000
33	c.1732-2 A>T	1.000	0.000	0.000	0.997	0.003	0.000	1.000	0.000	0.000
34	**c.1852_1853 AA>GC**	**0.994**	**0.006**	**0.000**	**0.990**	**0.010**	**0.000**	**0.984**	**0.016**	**0.000**
100	c.1942 C>T	1.000	0.000	0.000	0.997	0.003	0.000	1.000	0.000	0.000
35	**c.1959 G>T**	**0.963**	**0.037**	**0.000**	**0.977**	**0.023**	**0.000**	**0.975**	**0.025**	**0.000**
36	c.2152 C>T	1.000	0.000	0.000	0.994	0.006	0.000	1.000	0.000	0.000
	*MSH2*									
89	**c.-118 T>C**	**0.821**	**0.160**	**0.019**	na	na	na	**0.775**	**0.222**	**0.003**
87	c.131 C>T	1.000	0.000	0.000	1.000	0.000	0.000	1.000	0.000	0.000
88	c.134 C>T	1.000	0.000	0.000	1.000	0.000	0.000	1.000	0.000	0.000
39	c.212-23 A>C	1.000	0.000	0.000	1.000	0.000	0.000	1.000	0.000	0.000
90*	c.287 G>A	1.000	0.000	0.000	1.000	0.000	0.000	1.000	0.000	0.000
91*	c.329 A>G	1.000	0.000	0.000	1.000	0.000	0.000	1.000	0.000	0.000
92*	c.380 A>G	1.000	0.000	0.000	1.000	0.000	0.000	1.000	0.000	0.000
93	c.560 T>G	1.000	0.000	0.000	1.000	0.000	0.000	1.000	0.000	0.000
41	**c.965 G>A**	**0.960**	**0.040**	**0.000**	**0.944**	**0.056**	**0.000**	**0.974**	**0.026**	**0.000**
43	**c.1511-9 A>T**	**0.800**	**0.171**	**0.029**	**0.758**	**0.224**	**0.018**	**0.770**	**0.215**	**0.015**
48	c.1786_1788 del AAT	1.000	0.000	0.000	1.000	0.000	0.000	1.000	0.000	0.000
95*	c.2006-6 T>C/G	1.000	0.000	0.000	1.000	0.000	0.000	1.000	0.000	0.000
96	c.2062 A>G	1.000	0.000	0.000	1.000	0.000	0.000	1.000	0.000	0.000
97*	c.2139 G>C	1.000	0.000	0.000	1.000	0.000	0.000	1.000	0.000	0.000
50	c.2500 G>A	1.000	0.000	0.000	1.000	0.000	0.000	1.000	0.000	0.000
51	c.2542 G>T	1.000	0.000	0.000	1.000	0.000	0.000	1.000	0.000	0.000

* identified in CRC families from other populations than the Danish

^§ ^na: not analyzed

Variants that where polymorphic in sporadic CRC cohort or in the sub-cohort are in bold

**Table 4:** *The p*-values of marginal Fisher's exact tests between groups

Id	Variant	Sporadic-Sub-cohort	Familiar-Sub-cohort	Sporadic-familiar	All
	*MLH1*				
23	c.307-29 C>A	0.7259	1.0000	0.6673	0.8632
25	c.453+79 A>G	0.7908	-	-	-
24	c.350 C>T	-	0.2988	-	-
26	c.545+43 C>G	0.3263	1.000	1.000	0.4741
27	c.655 A>G	0.7715	0.5619	0.4869	0.7489
28	c.790+10 A>G	1.0000	-	-	-
29	C884+39G>A	-	0.2943	-	-
85	c.1217 G>A	1.0000	0.5359	0.4858	0.4833
31	c.1379 A>C	-	0.1880	-	-
32	c.1558+14 G>A	1.0000	0.6063	0.7685	0.8722
37	**c.1668-19 A>G**	0.5382	**0.0044**	0.0329	0.0148
33	c.1732-2 A>T	-	0.3109	-	-
34	c.1852_1853 AA>GC	0.2408	0.5672	0.6760	0.3569
100	c.1942 C>T	-	0.4747		
35	c.1959 G>T	0.2473	1.0000	0.3642	0.4493
36	c.2152 C>T	-	0.0840	-	-
					
	*MSH2*				
89	**c.-118 T>C**	**0.0037**	-	-	-
41	c.965 G>A	0.2574	0.0244	0.3609	0.0582
43	c.1511-9 A>T	0.0958	0.7314	0.0796	0.1617

The Bonferroni corrected *p*-value of sporadic versus sub-cohort is 0.05/13 = 0.0038

The Bonferroni corrected *p*-value of familiar-versus sub-cohort is 0.05/15 = 0.0033

Polymorphic variants with borderline significant *p*-values are in bold

Twenty-two of the variants analysed in the present study were neither detected in the sporadic cases nor in the sub-cohort (Tables 2 and 3). Among those were the six variants originally identified in other Caucasian populations i.e. hMLH1 c.1558+11 G>A and MSH2 c.287 G>A, c.329 A>G, c.380 A>G, c.2006-6 T>C/G and c.2139 G>T (see Table 2 for references). The remaining 16 variants, which have all been identified in Danish individuals with a familiar accumulation of CRC, are therefore very rare variants in the Danish population. Some of these rare variants have been classified either as neutral variants (e.g. *hMLH1 *c.2152 C>T and *hMSH2 *c.2500G>A) or pathogenic mutations (e.g. *hMLH1 *c.350 C>T, and *hMSH2 *c131 C>T) in Danish HNPCC families and/or HNPCC families from other populations (see Table 2 for references). To our knowledge, some of the rare variants have never been described previously neither in the Danish population nor in other populations (Table 2). These variants are most likely private variants found only in the families where they were originally identified. Four of the rare unclassified variants are missense variants changing either non-conserved amino acids i.e. *hMLH1 *c.1379 A>C and c.1689 A>G or conserved amino acids i.e. *hMSH2 *c.2062 A>G and c.2542 G>T.

### Linkage disequilibrium

**Figure 1:**
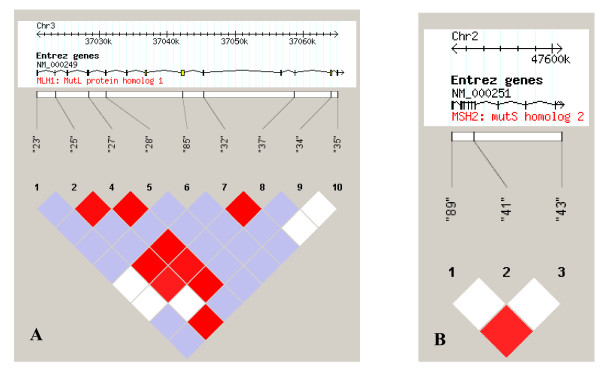
**LD plots of the sub-cohort in the genomic regions covering *hMLH1 *(A) and *hMSH2 *(B)**. Each square indicates the level of LD between two variants. The colours are defined as: red (high LD), LOD ≥ 2 and D' = 1; blue, LOD<2 and D' = 1; white, LOD<2 and D'<1. The top of the figure shows the genomic position and the known genes in the region.

Linkage disequilibrium (LD) analyses of the genomic regions covering *hMLH1 *and *hMSH2 *demonstrated high LD in the genomic regions covering the two genes. LD plots of the sub-cohort in the genomic regions covering the two genes are shown in Figures 1A and 1B. The nine polymorphic variants in *hMLH1 *in the sub-cohort are scattered along the whole genomic region of the gene. Only three polymorphic variants were identified in *hMSH2*. Two of these variants demonstrated high LD. They are positioned widely apart and thus cover a large area of the genomic region of *hMSH2*. The low LD of the third polymorphic variant in *hMSH2 *might be due to the low minor allele frequency of the variant. The high LD in the genomic regions covering the two genes indicates that common genetic variants in *hMLH1 *and *hMSH2 *in general are not involved in the development of sporadic CRC in the Danish population.

### *In silico *characterization of the variants

*In silico *functional characterization of the included missense variants was performed using SIFT [27], PolyPhen [28] and PMUT [29]. Nine of the missense mutations had previously been characterized at the functional level using different *in vitro *assays (references in Table 5). To test the accuracy of the *in silico *functional predictions we compared the results of the *in silico *analyses with the results of the *in vitro *functional analyses (Table 5). Concordance between predictions of all the *in silico *algorithms and the results of the *in vitro *functional analyses were found for 3/9 missense variants i.e. MLH1 p.Val219Ile, p.Ser406Asn and p.Pro648Ser. None of the *in silico *algorithms were more accurate in their predictions than the others when compared to the results of the *in vitro *functional analysis. The predictions of the SIFT and PolyPhen algorithms were frequently similar and often differed from the prediction made by PMUT. The above results demonstrate the importance of the use of *in vitro *functional analysis for the characterization of missense variants identified in individuals with hereditary CRC such as HNPCC.

**Table 5:** *In silico *functional characterization of the missense variants

Id	Variant	PMUT	SIFT	PolyPhen	Phylogeny	Activity in functional assays	References
	MLH1						
24	p.Thr117Met	Neutral	Not tolerated	Possible damaging	Conserved	Aberrant	[11,12]
27	p.Val219Ile	Neutral	Tolerated	Benign	Ile other species	Normal	[11,12]
85	p.Ser406Asn	Neutral	Tolerated	Benign	Non-conserved	Normal	[11]
**31**	**p.Glu460Ala**	Pathogen	Tolerated	Benign	Non-conserved	NA	-
**101**	**p.Ile563Met**	Neutral	Tolerated	Possible damaging	Non-conserved	NA	-
34	p.Lys618Ala	Pathogen	Not tolerated	Possible damaging	Non-conserved	Normal/aberrant	[9,11,45–47]
100	p.Pro648Ser	Pathogen	Not tolerated	Probably damaging	Conserved	Normal (aberrant protein stability)	[9,67]
36	p.His718Tyr	Neutral	Not tolerated	Probably damaging	Conserved	Normal	[11]
							
	MSH2						
89	p.Thr44Met	Neutral	Not tolerated	Possible damaging	Conserved	NA	-
87	p.Ala45Val	Neutral	Tolerated	Benign	Val other species	NA	-
90	p.Arg96His	Pathogen	Tolerated	Probably damaging	Non-conserved	NA	-
91	p.Lys110Arg	Neutral	Tolerated	Benign	Non-conserved	NA	-
92	p.Asn127Ser	Neutral	Not tolerated	Probably damaging	Conserved	NA	-
93	p.Leu187Arg	Neutral	Not tolerated	Probably damaging	Conserved	NA	-
41	p.Gly322Asp	Neutral	Tolerated	Benign	Conserved	Slightly reduced	[8,44]
**96**	**p.Met688Val**	Neutral	Not tolerated	Probably damaging	Conserved	NA	-
50	p.Ala834Thr	Pathogen	Tolerated	Possible damaging	Non-conserved	Normal	[10]
**51**	**p.Ala848Ser**	Neutral	Not tolerated	Possible damaging	Conserved	NA	-

NA, not available

Danish variants that have not been described previously are in bold

**Figure 2:**
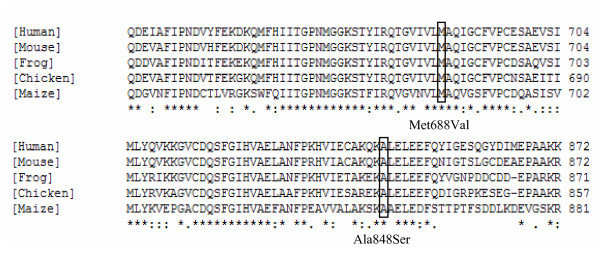
**Phylogeny of the MSH2 Met688Val and Ala848Ser variants**. Multiple sequence alignment of the hMSH2 polypeptide sequence and orthologues from other species were generated using the ClustalW algorithm [31]. The following polypeptide sequences were used in the alignment: P43246 (Human; *Homo sapiens*), CAA57049 (Mouse; *Mus musculus*), S53609 (Frog; *Xenopus laevis*), XP 426110 (Chicken; *Gallus gallus*) and CAB42554 (Maize; *Zea mays*).

There was no overall concordance between the *in silico *functional predictions of the four unclassified Danish variants i.e. MLH1 p.Glu460Ala, and p.Ile563Met and MSH2 p.Met688Val and p.Ala848Ser (Table 5.). The MLH1 p.Glu460Ala variant changes a non-conserved amino acid situated in the central region of MLH1 (Table 5). No defined functional domain has been assigned to this region of the protein [34]. The MLH1 p.Ile563Met variant changes a non-conserved amino acid situated in the PMS2 interaction domain of MLH1 (Table 5) [34]. The two variants in MSH2 i.e. p.Met688Val and p.Ala848Ser result in the elimination of conserved amino acids in the Walker A motif, required for ATP binding and the MutL homologue interaction domain, respectively (Table 5 and Figure 2) [35]. All the above characteristics of the missense variants indicate that at least the two missense variants in MSH2 might be disease causing mutations. However, further functional analyses are needed before any final conclusions can be drawn.

The putative role of all 35 variants in pre-mRNA splicing was analyzed using SNAP [30]. These analyses showed that several of the variants potentially either abolish or introduce Exon Splicing Enhancers (ESEs)(data not shown).

## Discussion

Identification of missense, silent and non-coding variants in genes involved in hereditary diseases always raises the intriguing question whether these variants are the disease causing mutations in the family/families where they are identified. Alternatively, they may be common variants causing a slight increase in sporadic disease susceptibility in the general population or simple neutral variants that are not involved in disease development. Missense, silent and non-coding variants are identified frequently in MMR genes (e.g. *hMLH1 *and *hMSH2*) in families fulfilling the Amsterdam II criteria. Functional analyses have shown that some of the missense variants identified in *hMLH1 *and *hMSH2 *result in reduced MMR activity and it has therefore been suggested that the decreased efficiency of DNA MMR could lead to increased cancer susceptibility [8-11].

In the present study, we have used a case-cohort design to elucidate the possible association between 35 variants in *hMLH1 *and *hMSH2*, either individually or as pairs, and the risk of sporadic CRC in the Danish population. The Danish Diet, Cancer and Health (DCH) cohort is large, population based and genetically homogeneous [20]. The sub-cohort used in the comparisons was selected randomly from the same cohort that gave rise to the cases with sporadic CRC; selection bias is thus unlikely. The cases and the individuals in the sub-cohort have been in the study equally long time and have approximately the same mean age (Table 1). We estimated that our study would be able to detect a disease susceptibility locus with an odds ratio (OR) of 2.5 with a power of 0.82, if the frequency of the disease causing genotype is 0.05, and a power of 0.77 if the OR is 2.0 and the disease causing genotype has a frequency of 0.1. Consequently, common disease causing alleles of moderate effect would most likely not be missed in the present study. To our knowledge, the frequencies of a large number of *hMLH1 *and *hMSH2 *variants and their association with sporadic CRC have never been analyzed in a large well defined Caucasian population. A previously published case-control study have demonstrated no association between MMR gene variants and the susceptibility to sporadic ovarian cancer [36]. In addition, previous association studies performed in Chinese and Korean populations did not find any association between variants in MMR genes and sporadic CRC [17,18]. The MLH1 c.415 G>C (p.Asp132His) variant has been shown to be associated with susceptibility to sporadic CRC in an Israeli population, although the CRCs associated with the variant usually are not MSI [19]. However, the p.Asp132His variant was present but not associated with sporadic CRC in a Chinese population [17]. Furthermore, the variant was not detected in a population of more 1,100 Americans with HNPCC-related cancers [37]. The p.Asp132His variant was not polymorphic in the Danish cohort of familiar CRC analyzed in the present study (data not shown). The frequency of the variant was not analyzed in the cohort of cases with sporadic CRC nor in the sub-cohort. However, due to high LD in the genomic region of *hMLH1 *this variant, if present in the Danish population, is most likely not associated with sporadic CRC.

Thirteen variants were polymorphic in the present study. The majority of the polymorphic variants (i.e. 9/13) were silent or present in non-coding regions. This corresponds with common variants being more abundant in introns and other regions than in coding regions [38]. Three of polymorphic variants i.e. MLH1 p.Val219Ile, p.Ser406Asn and p.Lys618Ala changed non-conserved amino acids and one i.e. MSH2 pGly322Asp changed a conserved amino acid. Furthermore, two polymorphic variants i.e.*hMSH2 *c.-118 T>C and *hMLH1 *c.1668-19 A>G demonstrated borderline significant *p*-values i.e. 0.0037 (significance level: 0.05/13 = 0.0038) and 0.0044 (significance level: 0.05/15 = 0.0033), respectively. It has been stated that the conventional threshold for declaration of statistical evidence of association (i.e. a *p*-value of 5 × 10^-2^) is not sufficient to detect gene-disease interactions. Consequently, it has been suggested to lower the threshold to 5 × 10^-5 ^[39]. The borderline significant *p*-values and the previously published functional analysis of one of the variants suggest that *hMSH2 *c.-118 T>C and *hMLH1 *c.1668-19 A>G are not associated with increased susceptibility to neither sporadic nor familiar CRC in the Danish population. The remaining polymorphic variants were not associated with CRC in the analyzed cohorts, neither individually nor as pairs, and they are hence neutral variants in the Danish population. Some of these variants i.e. *hMLH1 *c.655 A>G (p.Val219Ile), c.1217G>A (p.Ser406Asn), c.1558 +14 G>A and *hMSH2 *c.1959G>T (p.Leu653Leu) have been characterized as neutral variants in other studies as well [40-42]. *In vitro *functional analysis have also demonstrated that the variants MLH1 p.Val219Ile and p.Ser406Asn exhibit wild-type activity [8,11,12]. The *hMSH2 *variant c.965G>A (p.Gly322Asp) has previously been classified as a neutral variant [43]. This variant changes a conserved amino acid and functional analysis in yeast has shown that it has a slightly reduced MMR activity [8,44]. In the present study, the frequency of the c.965 G>A variant was higher in the cohort of individuals with familiar CRC compared to the sub-cohort although this was not statistically significant (Tables 3 and 4). Results regarding the MLH1 p.Lys618Ala variants have so far been contradictory. Functional analyses have identified this variant both as a pathogenic mutation and as a neutral variant [9,11,45-47]. Raevaara et al. used four different assays to evaluate the pathogenic status [9]. In all four assays the p.Lys618Val variant functioned like wt MLH1. In addition, p.Lys618Ala has been identified in one healthy control in a previous study [48]. The identification of the p.Lys618Ala variant in controls in the present study further support, that this variant is not a disease causing mutation involved in HNPCC or HNPCC-related cancers. In addition, the frequencies of the variant in the cohorts analyzed in the present study did not differ significantly and therefore p.Lys618Ala does not increase susceptibility to sporadic CRC in the Danish population. In conclusion none of the polymorphic variants in the present study are highly associated with CRC in the Danish population.

Linkage disequilibrium (LD) analysis showed high LD in the genomic regions covering *hMLH1 *and most of the genomic region covering *hMSH2 *in the Danish population. We therefore conclude that common variants in the two genes in general are not associated with susceptibility to sporadic CRC in a Danish population.

The age of the cohort of cases with sporadic CRC is relatively young (mean ~58 years) compared to the mean age of onset of colorectal CRC in the general Danish population (mean ~70 years) [49]. Consequently, the results obtained in the present study do not rule out that variants in *hMLH1 *and *hMSH2 *might be associated with sporadic colorectal cancer at an older age. However, generally cancers reflecting an inherited susceptibility seem to occur in a relatively young age.

Using *in silico *analysis several of the analyzed variants were predicted to either abolish or introduce ESEs (data not shown). It has, however, been shown that only a minor fraction of the variants predicted to change ESEs in *hMLH1 *and *hMSH2 *using *in silico *analysis do indeed change the pre-mRNA splice pattern *in vivo *[14,15]. In the study by Auclair et al., only ESEs at or close to 5' splice sites were found to cause aberrant splicing. None of the variants analysed in the present study affected 5' splice sites. Nevertheless, no final conclusions regarding splicing can be drawn from the *in silico *analyses.

Twenty two variants were not detected in the sporadic CRC cases and in the sub-cohort. Among those were the six variants originally identified more or less frequently in other populations (Table 2). All except one variant (c.380 A>G, p.Asn127Ser) performed very well in the SBE-tag array assay. This rules out that failure in detecting the variants caused their absence in the present cohorts. Consequently, these six variants are not polymorphic in the Danish population. The remaining variants were rare variants in the Danish population. They were only detected in individuals with familiar CRC and some of them have been classified as pathogenic mutations or neutral variants in other populations (see Table 2 for references). Four rare missense variants i.e. *hMLH1 *c.1379 A>C (p.Glu460Ala) and c.1689 A>G (p.Ile563Met) and, *hMSH2 *c.2062 A>G (p.Met688Val) and c.2542 G>T (p.Ala848Ser) were unclassified and have to our knowledge never been described previously neither in the Danish population nor in other populations. Other disease causing mutations have been identified in the families harbouring the two *hMLH1 *variants changing non-conserved amino acids i.e. pGlu460Ala and p.Ile563Met (unpublished results) consequently, these variants are most likely neutral and not involved in HNPCC. However, no other disease causing mutations have been identified in the families harbouring the *hMSH2 *variants changing conserved amino acids i.e. p.Met688Val and p.Ala848Ser variants. Further characterization of these variants such as functional studies and segregations analysis are needed before any final conclusion can be drawn regarding their pathogenicity.

In conclusion, high penetrance cancer susceptibility genes involved in hereditary syndromes have rarely emerged as definitive low-penetrance genes as a result of common variants increasing disease susceptibility [19]. The results shown in the present study demonstrate that the high-penetrance HNPCC genes *hMLH1 *and *hMSH2 *also do not appear to be low penetrance genes involved in sporadic CRC in the Danish population.

## Conclusion

More than half of the analysed variants in *hMLH1 *and *hMSH2 *were not polymorphic in the analysed cohort. The position of some of these rare variants in conserved regions of *hMSH2 *might indicate an association to the development of colorectal cancer in the families where they were identified originally. This hypothesis needs to be investigated further, using segregation and functional analysis. None of the polymorphic variants were highly associated with CRC in the Danish population. In addition, we demonstrated high LD in the genomic regions covering the two genes. Consequently, we conclude that common genetic variants in *hMLH1 *and *hMSH2 *in general are not involved in the development of sporadic CRC in the Danish population.

## Competing interests

The authors declare that they have no competing interests.

## Authors' contributions

LLC: Participated in the design of the study, carried out the genotyping using SBE-tag arrays and coordinated and drafted the manuscript. BEM: Carried out the statistical analyses and assisted in drafting the manuscript. FPW: Participated in the design of the study and assisted in drafting the manuscript. CW: Carried out the statistical analyses and assisted in drafting the manuscript. KK: Participated in the design of the study and the set-up of the SBE-tag arrays. AT: Responsible for the Danish Diet, Cancer and Health study. AO: Participating in the Danish Diet, Cancer and Health study. A–CS: Designed the SBE-tag array analysis for genotyping of the variants, CLA: Participated in the design of the study and assisted in drafting the manuscript. TFØ: Participated in the design of the study and assisted in drafting the manuscript. All authors read and approved the final version of the manuscript

## Pre-publication history

The pre-publication history for this paper can be accessed here:


